# An economic evaluation of implementing a decentralized dengue screening intervention under the National Vector Borne Disease Control Programme in Tamil Nadu, South India

**DOI:** 10.1093/inthealth/ihab045

**Published:** 2021-08-28

**Authors:** Malaisamy Muniyandi, Nagarajan Karikalan, Karunya Ravi, Senthilkumar Sengodan, Rajendran Krishnan, Kirti Tyagi, Kavitha Rajsekar, Sivadhas Raju, T S Selvavinayagam

**Affiliations:** Scientist-D & Head, Department of Health Economics, ICMR-National Institute for Research in Tuberculosis, No. 1, Sathyamoorthy Road, Chetpet, Chennai 60003, India; Scientist-D & Head, Department of Health Economics, ICMR-National Institute for Research in Tuberculosis, No. 1, Sathyamoorthy Road, Chetpet, Chennai 60003, India; Scientist-D & Head, Department of Health Economics, ICMR-National Institute for Research in Tuberculosis, No. 1, Sathyamoorthy Road, Chetpet, Chennai 60003, India; Scientist-D & Head, Department of Health Economics, ICMR-National Institute for Research in Tuberculosis, No. 1, Sathyamoorthy Road, Chetpet, Chennai 60003, India; Scientist-D & Head, Department of Health Economics, ICMR-National Institute for Research in Tuberculosis, No. 1, Sathyamoorthy Road, Chetpet, Chennai 60003, India; Department of Health Research, Ministry of Health and Family Welfare, 2nd Floor, IRCS Building, 1, Red Cross Road, New Delhi 110001, India; Department of Health Research, Ministry of Health and Family Welfare, 2nd Floor, IRCS Building, 1, Red Cross Road, New Delhi 110001, India; Department of Public Health and Preventive Medicine, Government of Tamil Nadu, 359, Anna Salai, Chokkalingam Nagar, Teynampet, Chennai 600006, India; Department of Public Health and Preventive Medicine, Government of Tamil Nadu, 359, Anna Salai, Chokkalingam Nagar, Teynampet, Chennai 600006, India

**Keywords:** cost-effectiveness, dengue, economic evaluation, QALY, screening

## Abstract

**Background:**

Lack of effective early screening is a major obstacle for reducing the fatality rate and disease burden of dengue. In light of this, the government of Tamil Nadu has adopted a decentralized dengue screening strategy at the primary healthcare (PHC) facilities using blood platelet count. Our objective was to determine the cost-effectiveness of a decentralized screening strategy for dengue at PHC facilities compared with the current strategy at the tertiary health facility (THC) level.

**Methods:**

Decision tree analysis followed a hypothetical cohort of 1000 suspected dengue cases entering the model. The cost-effectiveness analysis was performed at a 3% discount rate for the proposed and current strategy. The outcomes are expressed in incremental cost-effectiveness ratios (ICERs) per quality-adjusted life years gained. One-way sensitivity analysis and probabilistic sensitivity analysis were done to check the uncertainty in the outcome.

**Results:**

The proposed strategy was found to be cost-saving and ICER was estimated to be −41 197. PSA showed that the proposed strategy had a 0.84 probability of being an economically dominant strategy.

**Conclusions:**

The proposed strategy is cost-saving, however, it is recommended to consider optimal population coverage, costs to economic human resources and collateral benefits of equipment.

## Introduction

Dengue is the most common vector-borne infection globally, with an estimated 100–400 million infections occurring every year.^1^ There is no effective vaccine or medicine available to prevent or cure dengue and it leads to around 20 000 deaths per year.^2^ Symptomatic dengue infections have a broad range of severity and the clinical characteristics of dengue may mimic other disease characteristics, which increases the probability of misdiagnosis.^3^

India contributes around 34% of the global burden of dengue.^4^ A meta-analysis of Indian studies estimated the dengue fatality rate to be 2.6%.^5^ Although dengue is a notifiable disease in India, studies and modelling estimates suggest that the disease is grossly underreported due to the existing gaps in the public health surveillance system. Dengue surveillance in India is conducted through a network of >600 sentinel hospitals under the National Vector-Borne Disease Control Programme (NVBDCP), Integrated Disease Surveillance Program (IDSP) and a network of 52 Virus Research and Diagnostic Laboratories (VRDLs).^6^ Tamil Nadu (TN) is one of the largest states in India and reported a high burden of dengue infection.^7^ High dengue disease burden and frequent outbreaks result in an adverse impact on the country's economy and strain the health system.

One of the major hindrances in the control and management of dengue infection is the lack of timely and point-of-care diagnosis in TN. The complex clinical presentation of dengue symptoms and a lack of effective screening and diagnosis results in delayed diagnosis and leads to rapid disease progression and mortality. Dengue diagnosis and management in India is undertaken as per NVBDCP^8^ guidelines that recommend screening of platelet counts and enzyme-linked immunosorbent assay (ELISA) testing for confirmation. Currently both screening and confirmatory testing for a dengue diagnosis are being done only at the tertiary healthcare (THC) facilities in TN, which requires multiple visits. This results in an increased patient load at the THC facilities and remains a barrier for access by all sections of the population. It also leads to increased out-of-pocket expenditures for patients, delay in diagnosis, delay in treatment and loss to follow-up for treatment.

To address this gap in the dengue diagnostic cascade, the government of TN has adopted a decentralized dengue screening strategy at the primary healthcare (PHC) facilities. In this strategy, haematology analyser equipment is installed at the PHC facilities for screening suspected dengue cases with low platelet count (thrombocytopenia) who will be further referred to a THC facility for confirmation and disease management. The strategy of using platelet counts (thrombocytopenia) for predicting dengue has been increasingly recognised in the research literature as a valid screening tool. Studies have shown the importance of haematology analysers in hospital-based settings with a minimal patient load.^9,10^ This strategy prioritizes the present delay in diagnosing dengue at an earlier stage, which could help reduce dengue morbidity and mortality. However, the implementation of such a screening strategy has not been studied for its suitability in large community settings with a high dengue burden. In the absence of any large-scale primary evaluation studies, we utilised economic modelling to provide evidence for policymakers to implement evidence-based interventions. Thus the objective of this study was to determine the cost-effectiveness of a decentralized screening strategy for dengue at PHC facilities compared with the current strategy at THC facilities.

## Methods

This economic evaluation was conducted by following the Consolidated Health Economic Evaluation Reporting Standards guidelines (eTable 1). This is a model-based study from a societal perspective for the year 2019.

### Study setting and population

The study was conducted in TN, where the decentralized screening strategy has been implemented at the district level. Further, this strategy is being considered for scale-up at the subdistrict level in a phased manner. Our study population represents a hypothetical cohort of 1000 individuals who access care at PHC facilities in these settings with fever and symptoms suggestive of dengue.

### Comparator and time horizon

The present model compares the proposed decentralized dengue screening strategy at the PHC facilities with the current screening strategy used for dengue diagnosis at the THC facilities. Under the decentralized strategy, point-of-care screening is provided for those who access PHC facilities with fever and symptoms suggestive of dengue. Both the strategies involve a confirmatory ELISA test for dengue at the THC facilities. A single episode of dengue infection with a lifetime horizon and a global discount rate of 3% is considered for the estimation of effectiveness and cost.

### Model assumption

This economic model was conceptualized based on the natural history of dengue infection followed by ambulatory or hospitalization-based treatment and care. This model characterises all the health states of dengue from infection to cure or death. The specific assumptions used for the model were two repeat dengue screening tests at 2-d intervals followed by an ELISA test for those with a platelet count (PLC) >100 000/mm^3^ in the proposed strategy, one repeat dengue screening test at a 2-d interval with an ELISA test for those with a PLC >100 000/mm^3^ in the current strategy, a 50% reduction in severe dengue cases due to early diagnosis in the proposed strategy (based on expert opinion) and no loss to follow-up in the screening, diagnosis and treatment cascade (considered in both strategies, as there are no available data on loss-to-follow-up details).

### Model structure

The present study utilised an economic model to calculate the incremental cost-effectiveness ratios (ICERs) for the current and proposed dengue screening strategies. The ICER is calculated from the cost and quality-adjusted life years (QALYs) of the two different strategies for dengue screening. The strategies considered for dengue diagnosis are summarized in Table 1.

**Table 1.** Different screening strategies for dengue

Strategies	Level of implementation	Diagnostic tool	Population	Frequency of screening	Referral
Proposed strategy	PHC	CBC for platelet level	Persons with febrile illness and warning signs	Repeat CBC at days 2 and 4 if PLC >100 000/mm^3^	Tertiary for ELISA. If ELISA is positive, repeat CBC twice a day and hospitalize
Comparator	THC	CBC and ELISA	Persons with febrile illness and warning signs	Repeat CBC once for self-reporting patients at 2-d intervals if PLC >100 000/mm^3^	If ELISA is positive, repeat CBC twice a day and hospitalize

### Current strategy

Patients with 2–5 d of febrile illness having two or more symptoms of headache, retro-orbital pain, myalgia, arthralgia, rash and haemorrhagic manifestations are considered as suspected dengue cases. The suspected dengue cases approaching the PHC facility were referred to the THC facility for diagnosis. At the THC facility, the symptoms are managed along with a dengue diagnosis. A dengue diagnosis in the THC facility is done by testing the PLC (<100 000/mm^3^) for screening and an ELISA test for confirmation. During treatment the patient is subjected to continuous monitoring at the THC facility for a reduction in the blood PLC.

### Proposed strategy

For early diagnosis of dengue, it is proposed to screen the suspected dengue cases at the PHC facility using a haematology analyser. The PLC is assessed and those with <100 000/mm^3^ will be referred to the THC facility for further management and those with >100 000/mm^3^ will be reassessed at 2-d intervals. A maximum of two repeat PLCs will be undertaken to rule out dengue.

### Decision tree model

**Figure 1.**
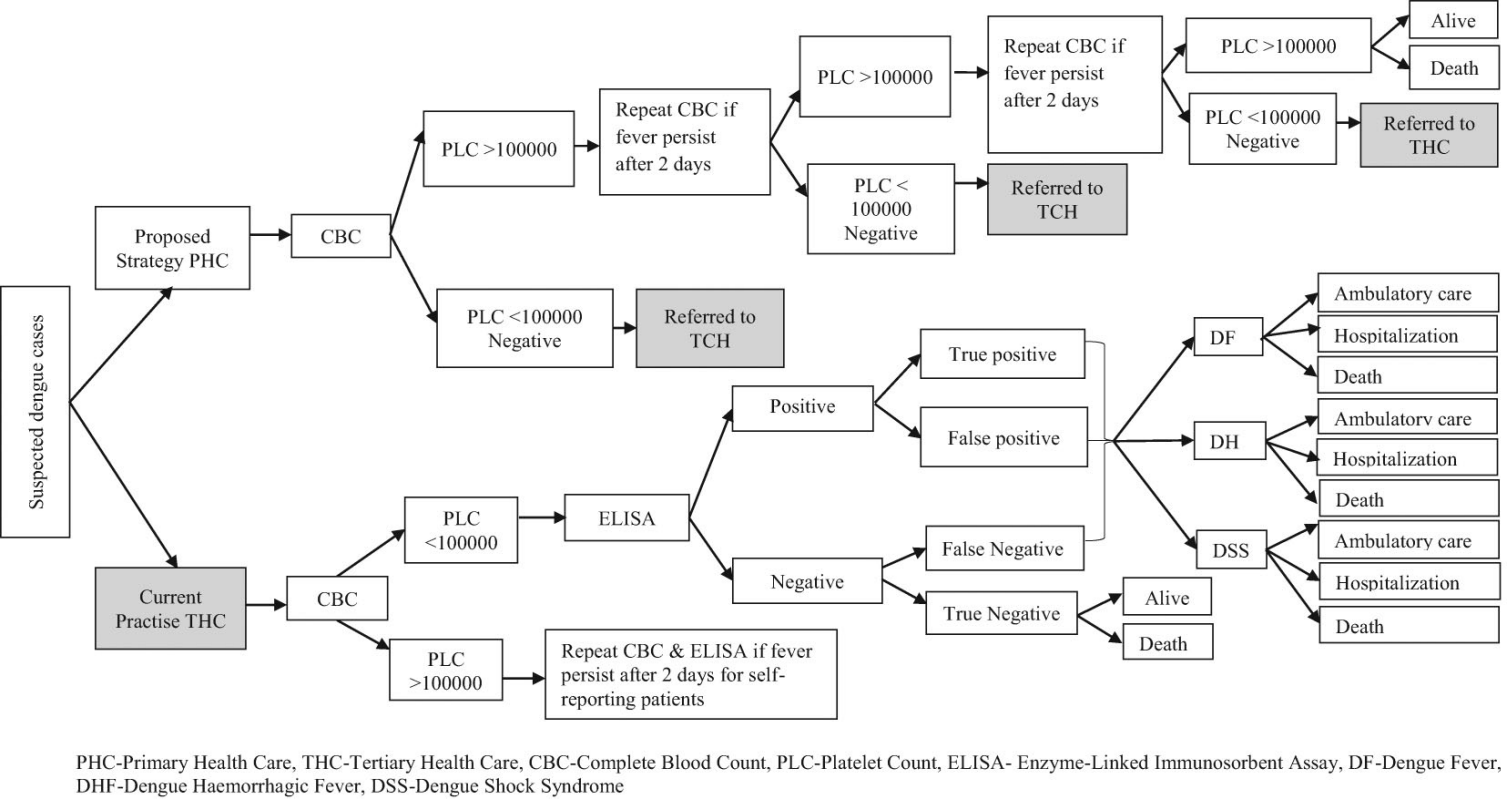
Decision tree for dengue screening at PHC facility as compared with THC facility.

The decision model planned for this study was constructed based on the natural progression of dengue and its diagnostic cascade (Figure 1).^7,11–13^ Both the proposed and current strategies were modelled as two parallel trees using probabilities associated with the dengue diagnosis, treatment and outcome. A patient diagnosed with dengue in each strategy was further classified based on the disease severity into three disease states: dengue fever (DF), dengue haemorrhagic fever (DHF) and dengue shock syndrome (DSS). In each disease state, the outcomes considered were survival after hospitalization, survival after ambulatory treatment and death. An Excel spreadsheet (Microsoft, Redmond, WA, USA) and TreeAge software (version 1.0; TreeAge Software, Williamstown, MA, USA) were used for analysis.

### Model input parameters

The key input parameters for the model include demography;^7^ prevalence of dengue;^5^ prevalence of suspected dengue cases;^14^ sensitivity and specificity of the ELISA;^4^ health state probability of DF,^15^ DHF,^14^ DSS^14^ and death due to dengue;^5^ all-cause mortality;^16^ probability of cure;^17^ quality-of-life score for different health states^18^ and health system and patient costs incurred for dengue diagnosis and treatment.^16,19^ All this information was extracted from the published literature and NVBDCP reports. Table 2 presents the input parameter values used in the analysis with 20% upper and lower limits along with their distribution.

**Table 2.** Input parameters used for model-based cost-effectiveness analysis of dengue screening at the PHC facility

	Parameters	To model	Lower limit	Upper limit	Distribution	Source
Demographic values	Average age of suspected dengue cases (years)	22	NA	NA	NA	5
	Life expectancy for an average age of suspected dengue cases (years)	53	42.4	63.6	Normal	15
	Cohort population	1000	NA	NA	NA	Assumption
Prevalence	Seroprevalence of dengue	0.383	NA	NA	NA	5
Mortality	Probability of all-cause mortality for the average age of suspected dengue cases	0.006	0.005	0.007	β	15
	Probability of death due to dengue in the current strategy	0.026	NA	NA	NA	5
	Probability of death due to dengue in early screening	0.010	NA	NA	NA	1
	Probability of death due to DF	0	0	0	NA	Assumption
	Probability of death due to DHF in the current strategy	0.010	0.008	0.012	β	Estimated
	Probability of death due to DSS in the proposed strategy	0.015	0.012	0.018	β	Estimated
	Relative risk of mortality due to DHF in the proposed strategy	0.380	0.300	0.460	β	Estimated
	Relative risk of mortality due to DSS in the proposed strategy	0.380	0.300	0.0460	β	Estimated
CBC	Probability of PLC <100 000/mm^3^ in the presence of warning signs	0.710	0.568	0.852	β	13
	Probability of CBC test positive	0.399	0.319	0.478	β	Estimated
ELISA	Sensitivity	0.77	NA	NA	NA	4
	Specificity	0.94	NA	NA	NA	4
	True positive	0.888	0.704	1	β	Estimated
	True negative	0.868	0.694	1	β	Estimated
Disease state probability	Lab confirmed DF in the current strategy	0.77	0.616	0.924	β	5
	Lab confirmed DHF in the strategy	0.18	0.144	0.216	β	5
	Lab confirmed DHF in the proposed strategy	0.09	0.072	0.108	β	Assumption
	Lab confirmed DSS in the current strategy	0.05	0.04	0.06	β	5
	Lab confirmed DSS in the proposed strategy	0.025	0.02	0.03	β	Assumption
	Outpatients among patients with DF	0.68	0.544	0.816	β	32
	Outpatients among patients with DHF	0.26	0.208	0.312	β	32
	Outpatients among patients with DSS	0	0	0	NA	32
Utility value	Utility for death	0	0	0	NA	By Definition
	Utility for undifferentiated fever	0.91	0.728	1	β	Assumption
	Utility for DF	0.91	0.728	1	β	Assumption
	Utility for DHF	0.66	0.528	0.792	β	17
	Utility for DSS	0.41	0.328	0.492	β	Assumption
Diagnostic cost	Cost of CBC per test (in INR)	153.65	122.92	184.38	γ	18
	Cost of ELISA per test (in INR)	314.85	251.88	377.82	γ	18
Treatment	Cost of ambulatory not-fatal disease per case (in INR)	2896.78	2317.42	3476.13	γ	16
	Cost of hospitalized not-fatal disease per episode (in INR)	21 816.20	17 452.96	26 179.44	γ	16
	Non-medical cost per non-fatal case in the current strategy (in INR)	1260.20	1008.16	1512.24	γ	16
	Non-medical cost per non-fatal case in the proposed strategy	630.10	504.08	756.12	γ	Assumption
	Direct fatal cost per case	5186.11	4148.88	6223.33	γ	16
	Indirect fatal cost per case	2 730 021.97	2 184 017.56	3 276 026.35	γ	16
Willingness-to-pay threshold	Willingness-to-pay threshold (GDP per capita, in INR)	135 966	–	–	NA	19

NA: not applicable.

### Cost data

Cost data were used from a meta-analysis report of Indian studies.^16^ The average cost of dengue non-fatal and fatal cases was estimated, which included health system costs, patient out-of-pocket expenditures and productivity loss. Information on health system costs such as complete blood count (CBC) and ELISA costs were obtained from the Central Government Health Scheme (CGHS) rates for 2014 published by the government of India. All the costs were adjusted for 2019. The total cost incurred by a dengue patient for a single disease episode was calculated separately in each strategy.

### Effectiveness data

Health outcomes are determined in terms of the number of suspected dengue cases diagnosed with dengue infection and their related quality-of-life scores. Since the utility score for each state of dengue infection was not available for India, we used contextually relevant data from Indonesia, a comparable Asian country.^17^ Treatment outcomes in terms of cure and mortality were taken from an Indian meta-analysis report.^5^

### Other data

Life expectancy was taken from India's life table, published based on Sample Registration System (SRS) data.^15^ The start age of the cohort in the model was 22 y, which was based on the mean age of dengue patients during the time of diagnosis. The mean age of dengue infection and prevalence of dengue were used from the recently published meta-analysis on dengue infection in India.^5^

### Base case analysis

Estimation of incremental costs and QALYs gained by the introduction of a new dengue screening strategy for the cohort population entering the decision-analytic model was done. Results were expressed in terms of QALYs and life years gained, deaths averted and cost per cure.

### Calibration and sensitivity analysis

The robustness of model results was tested through sensitivity analysis by varying input parameters between 20% above and below the estimated values. The parameter uncertainties that could influence the ICER were evaluated by one-way sensitivity analysis (OWSA) and presented in a tornado diagram. The robustness of the model was further evaluated by probabilistic sensitivity analysis (PSA), which gives 1000 different costs and effectiveness for each strategy. The resulting ICER values were plotted in a scatter plot. The cost-effectiveness acceptability curve (CEAC) was drawn to indicate the model's probabilistic response to different cost-effectiveness thresholds.

### Budget impact analysis

The costs of the haematology analyser, reagents, training and maintenance were considered for implementation costs. The unit cost for implementing the screening strategy at the PHC facility and the estimated population coverage were used to calculate the additional budget requirement of the government of TN for implementing haematology analysers for screening suspected dengue cases at the PHC facility. Assuming increased coverage of suspected dengue cases in the PHC over years, the cost, QALY and ICER values were estimated. The projection was made for 5 y with 10–70% coverage compared with the base year.

### Model outcomes

The outcomes of the model were expressed in terms of QALYs and life years gained, including the overall cost incurred per patient in intervention and comparator strategies. The model also compared the incremental cost with incremental QALYs to obtain the ICER. Further, the ICER was compared with a threshold of one-time gross domestic product (GDP) per capita in India to determine its cost-effectiveness.^20^ We also calculated net monetary benefit (NMB) and the additional budget required for implementing haematology analysers for screening suspected dengue cases at the PHC facilities.

## Results

### Model descriptive analysis

The average age of the suspected dengue cases entering the cohort was 22 y (Table 3). The proposed decentralised screening strategy detected 24 additional dengue cases when compared with the current strategy. Severe dengue cases and deaths (three vs four) were reduced in the proposed strategy as compared with the current strategy. The hospitalization for dengue was more in the proposed strategy as compared with the current strategy (12.05% vs 11.1%).

**Table 3.** Results of model descriptive analysis for dengue screening at PHC facility

Parameters, n (%)	Proposed strategy	Current strategy
Test positive	130 (26)	106 (21)
DF	146 (29)	103 (21)
DHF	15 (3)	24 (5)
DSS	4 (0.82)	7 (1.34)
Hospitalized care	60 (12.05)	56 (11.12)
Outpatient care	103 (20.60)	77 (15.38)
Death	3 (0.65)	4 (0.79)

### Base case analysis

#### Cost estimation

The total health system cost for the cohort estimated in the proposed and current strategies were INR1 910 131 and INR1 696 177, respectively (Table 4). It contributed 17.45% and 14.67% to the total cost estimated in the proposed and current strategies, respectively. Patient out-of-pocket expenditures and productivity loss were reduced in the proposed strategy when compared with the current strategy (INR9 030 056 vs INR9 859 103). The productivity loss due to premature death contributed more to the total cost in the proposed and current strategies (81.57% and 83.87%, respectively).

**Table 4.** Summary of costs estimated for dengue screening at PHC facility

	Total cost (in INR)	
Cost type	Proposed strategy	Current strategy
Health system cost		
Ambulatory care cost (medical) for non-fatal cases	297 807	221 723
Hospitalization care cost (medical) for non-fatal cases	1 319 351	1 230 295
Investigation cost	273 997	223 556
Medical cost for death patients	18 977	20 602
Total health system costs	1 910 131	1 696 177
Patient costs		
Non-medical cost for non-fatal cases	102 884	167 525
Indirect cost for death	8 927 172	9 691 578
Total patient costs	9 030 056	9 859 103

The total cost incurred for the diagnosis and treatment of dengue in the current strategy was more when compared with the proposed screening strategy (Table 5). The undiscounted cost per person for the current and proposed strategies was INR23 564 and INR22 372, respectively. Discounting for 53 y of average remaining life expectancy, the estimated discounted cost per person was INR4919 and INR4670, respectively.

**Table 5.** Base case results for dengue screening at PHC facility

Parameters	Proposed strategy	Current strategy
Total costs (in INR)		
Undiscounted	11 186 000	11 782 000
Discounted	2 335 000	2 458 000
Total life years		
Undiscounted	26 327	26 312
Discounted	5496	5493
Total QALYs		
Undiscounted	23 665	23 455
Discounted	4940	4896

#### Effectiveness estimation

The total undiscounted life-years and QALYs estimated in the proposed strategy was more when compared with the current strategy (Table 5). The undiscounted QALYs per person for the current and proposed strategies was 46.91 and 47.33, respectively.

#### Incremental costs and effectiveness

The proposed strategy was cost-saving and the incremental cost saved over the current strategy was estimated to be INR1192 per person (Table 6). The total incremental life years gained with and without discount was 3.02 and 14.4, respectively, and the total incremental QALYs gained with and without discount was 43.83 and 210, respectively.

**Table 6.** Model outcome summary table for dengue screening at PHC facility

Outcome	Value
Incremental cost (in INR)	
Undiscounted	−596 000
Discounted	−124 415
LYs gained	
Undiscounted	14.4
Discounted	3.02
QALYs gained	
Undiscounted	210
Discounted	43.83
ICER (using LYs)	
Undiscounted	−41 388.88
Discounted	−41 197.01
ICER (using QALYs)	
Undiscounted	−2838.90
Discounted	−2838.58
Total death averted	0.27

#### ICER

**Figure 2.**
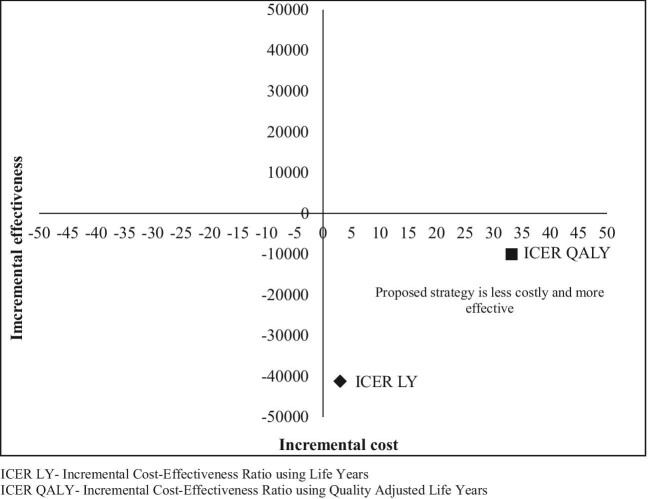
The cost-effectiveness plane for dengue screening at PHC facility as compared with THC facility.

The ICER calculated using discounted life years and QALYs gained was −41 197.10 and −2383.58, respectively (Table 6). The incremental cost-effectiveness plane indicated that the proposed dengue screening strategy was more effective and less expensive compared with the current strategy (Figure 2).

#### Out-of-pocket expenditures

In terms of out-of-pocket expenditures, the current screening strategy incurs INR64 641 additional costs than the proposed strategy. The proposed intervention increased case detection compared with the current strategy, however, due to reduced travel distance and visits to tertiary care facilities, patients saved INR129 per person.

#### NMB

The NMB estimated using the willingness to pay was INR66.9 million for the proposed strategy and INR66.3 million for the current strategy. This resulted in an incremental NMB of INR0.61 for the proposed strategy, suggesting that the proposed strategy was highly acceptable for achieving a net benefit.

#### OWSA

**Figure 3.**
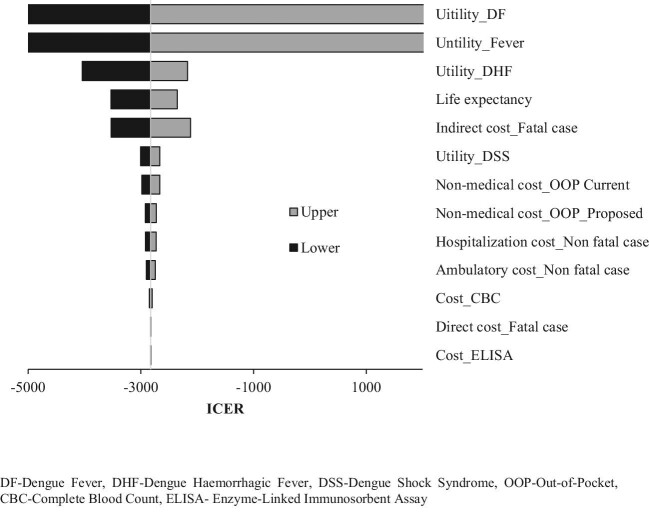
Tornado plot illustrating the effect of individual parameters on the ICER. OOP: out of pocket.

In the OWSA, the utility parameters of the DHF and DSS dengue health states had a greater influence on the ICER value (Figure 3).

#### PSA

**Figure 4.**
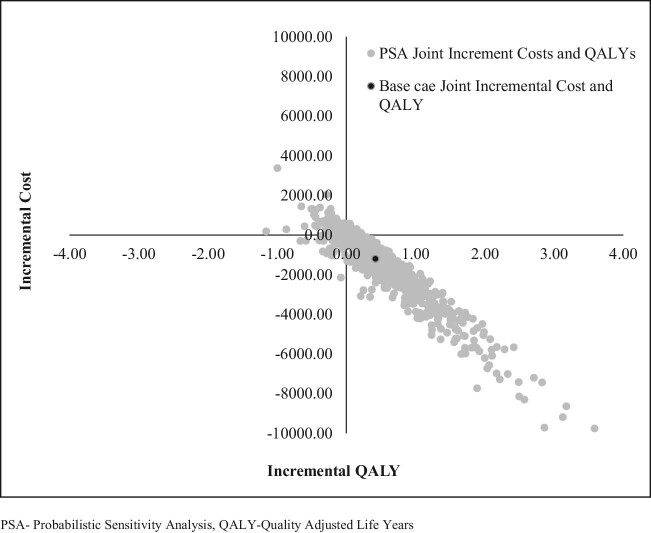
Incremental cost-effectiveness scatterplot in the PSA.

The PSA showed that the joint incremental cost and effectiveness using QALYs was less costly and more effective for approximately 84% of the iteration values (Figure 4).

#### CEAC

**Figure 5.**
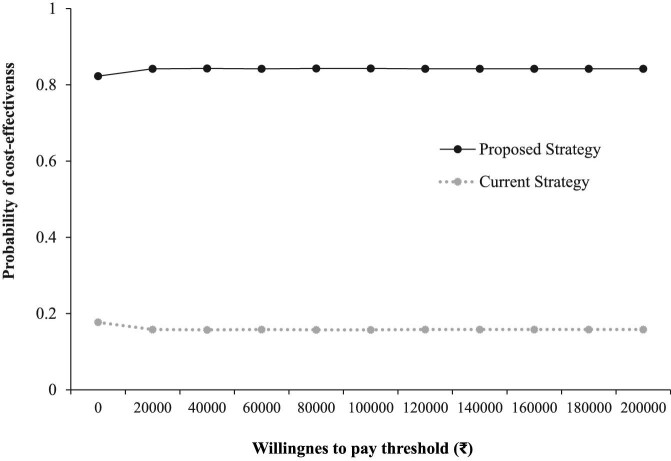
The CEAC.

The CEAC highlights that the proposed strategy had a 0.8 probability of being an economically dominant strategy as compared with the current strategy at different cost-effectiveness threshold values (Figure 5).

### Budget impact analysis

**Figure 6.**
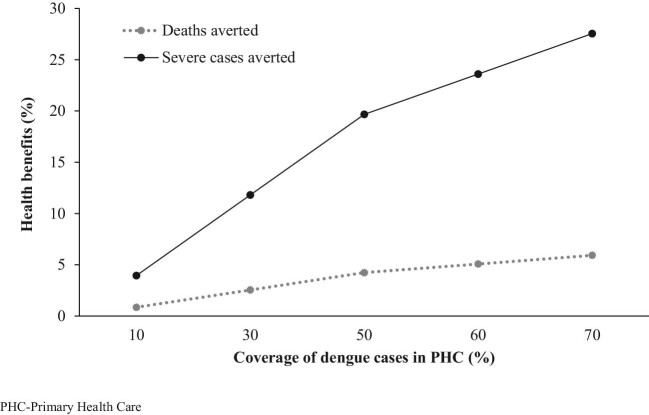
Health benefit based on population coverage for 5 y.

Based on 2011 census data, the projected population of TN for 2019 was 82 439 997. Considering a 14.8% prevalence of dengue acute febrile illness in TN and 0.38% seropositive prevalence, 46 365 dengue cases were estimated for the base year. An additional budget of INR57.4 million would be required for the government to implement the proposed screening strategy in the base year. With the increase in the proposed strategy's population coverage, deaths and severe dengue cases (DHF and DSS) averted increased (Figure 6).

#### ICER vs variations in PHC access for dengue screening

**Figure 7.**
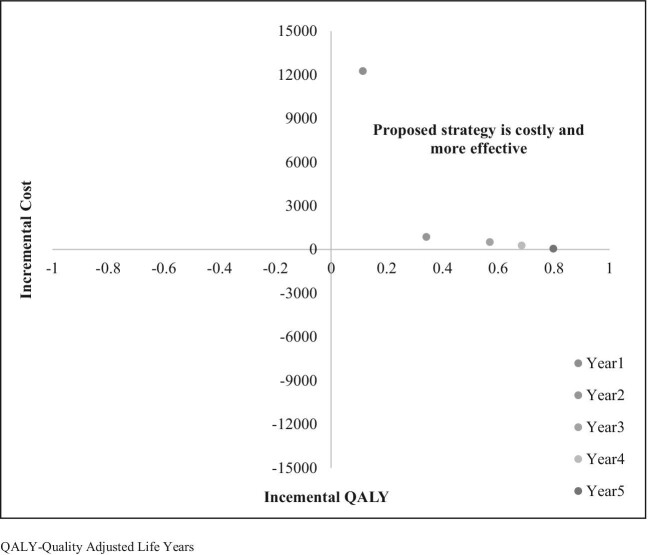
Incremental cost-effectiveness based on population coverage for 5 y.

Assuming a 10–70% increase in PHC access over 5 y, it was found that health benefits gained in terms of QALYs increased (Figure 7). Without implementation costs, the proposed strategy was found to be less costly and more effective even at 10% coverage in the PHC facilities. However, the cost was high in the implementation phase, which gradually decreased over years. Thus the ICER values considering implementation costs suggest that the proposed strategy was more costly and more effective.

## Discussion

We assessed the economic impact of using haematology analyser equipment at the PHC facilities for dengue screening. A decision tree model was used for this evaluation that compared the intervention with the current strategy. Dengue preventive measures such as vector control and vaccination are highly challenging. Model-based studies have been undertaken in high-burden settings to assess the cost-effectiveness of various control measures, including pre-screening for vaccination.^21,22^ These studies highlight that extensive resources are required for the large-scale implementation of such strategies, which makes it difficult to adopt in a resource-constrained setting. In the absence of dengue vaccination in the current study setting, early diagnosis of dengue and prompt care are the only effective ways to address the mortality and morbidity due to dengue. Still, the present decentralised approach for dengue screening proposed in TN could well be integrated with future vaccination efforts in high dengue burden settings. Such an integrated approach has proven to be useful to cater to the diagnostic and vaccine prevention needs of the population at risk of vector-borne diseases.^23,24^

Evidence suggests that dengue patients experience significant disability during the acute and hospitalization phase of the infection.^25^ There is also a significant decrease in quality of life in these patients during the hospitalization and recovery periods.^26^ The key findings from our evaluation highlight that the implementation of dengue screening at PHC facilities could lead to an incremental gain in QALYs and life years in dengue patients. Our study projected the health outcomes of dengue patients for their lifetime, which highlights that the interventions aimed at the early stage of diagnosis and acute phase of dengue could lead to long-term health gains.

Systematic reviews conducted on the economic benefits of the rapid diagnostic test (RDT) for dengue found that the RDT for dengue was cost effective in endemic settings.^27,28^ It was also found that the RDT was less advantageous to symptomatic treatment and management. The review emphasised the background burden of the setting to be determinant of cost-effectiveness and highlighted the limitations of generalising cost-effectiveness evidence. The present study was conducted within a high dengue burden state in India and the results can be generalized only to a similar setting.

A study in south India on the comparative evaluation of validity and cost–benefit analysis of the RDT reported that the RDT was useful in dengue-endemic and resource-limited settings for screening.^29^ Our economic evaluation within the same setting identified that the implementation of a dengue screening test at PHC facilities is cost effective, with additional gains in QALYs and a reduction in mortality. This was especially strongly associated with the level of access to diagnostic services at the PHC facilities.

The cost-effectiveness finding of the proposed screening strategy in the present evaluation needs to be interpreted in the context of decentralisation of diagnostic service at the PHC facilities. The importance of strengthening the PHC facilities for effective healthcare delivery in India was highlighted earlier. India has a three-tier health system and the urban–rural distribution of THC facilities is quite unequal. This results in almost two-thirds of patients in urban hospitals being from rural areas.^30^ Studies conducted on decentralised screening of infectious disease have highlighted the economic benefits associated with such strategies.^31–33^ Our study results are in line with these findings, suggesting that the decentralized dengue screening strategy was cost-saving.

The decentralised nature of our proposed diagnostic strategy was identified as a cost-saving intervention for both the health system and patients. The out-of-pocket expenditures experienced by patients was found to be decreased due to the proposed intervention. The cost-saving strategy could be due to early diagnosis followed by early treatment, resulting in the prevention of acute and prolonged illness due to delayed diagnosis. Our sensitivity analysis highlights that the three factors—QALYs of patients with DF and DHF health states and the cost of death due to dengue—had a greater influence on the ICER value. This underscores the possible impact of dengue infections on the long-term quality of life of individuals. The decentralised diagnostic strategy could improve early diagnosis and might result in an incremental gain in quality-of-life scores.^34^

Delayed diagnosis of dengue could potentially lead to hospitalisation and increased bed days and thus increase the expenditures for both the health system and the patient.^35^ Our study identifies a reduction in the severity of dengue cases, including DHF and DSS. This again could be attributed to the decentralized nature of the proposed dengue screening strategy. Considering the implementation costs, the proposed screening strategy was still cost effective. However, we did not account for the other collateral benefits of the equipment used for dengue screening at the PHC facility (Table 7). The current implementation plan of the state government does not prioritize the use of equipment for disease diagnosis other than dengue, consideration of which could reduce the cost further.

**Table 7.** Other benefits of haematology analyser at PHC facility

Disease	Haematology analysis
Typhoid and other non-specific fevers^36^	Lymphocyte count (<40%)PLC (150 001–450 000/mm^3^)
Malaria^37^	Haemoglobin (9.8 g/dl)
	PLC (50 000–100 000/mm^3^)
	Lymphocyte count (<40%)
Japanese encephalitis^38^	PLC (<50 000/mm^3^)
Antenatal care	Haemoglobin (monitoring normal range)
Neonatal sepsis^39^	Haematological scoring system (score >5)

### Limitations of the study

This economic evaluation model was conducted using published and secondary data. Also, the economic costs for human resources (HR) was not considered in this study. Further evaluation of this model is planned using primary data from the field, including the economic costs for HR. The utility score for dengue health states included in the model was utilized from a different setting and may have resulted in over- or underestimation of the benefits and ICERs. However, this limitation was addressed through the sensitivity analysis technique.

## Conclusions

The model results conclude that dengue screening at the PHC facility in TN is cost-saving and cost effective at 70% and 10% population coverage, respectively, when compared with the current strategy. Although high costs are incurred for the implementation of the intervention, our budget impact analysis highlights that the proposed strategy turns cost-saving from the fifth year of implementation.

## Supplementary Material

ihab045_Supplemental_File

## Data Availability

The authors have the research data, which are avail able upon reasonable request.
