# Supplementary material for: An economic evaluation of implementing a decentralized dengue screening intervention under the National Vector Borne Disease Control Programme in Tamil Nadu, South India
Source: Int Health. 2021 Aug 28;14(3):295–308. doi: 10.1093/inthealth/ihab045 (PMC9070504; doi:10.1093/inthealth/ihab045)
Supplement: ihab045_Supplemental_File [file ihab045_supplemental_file.docx]

**Technical Note**

**Defintions**

**Dengue suspects**

Patient with 2-5 days of febrile illness having two or more of the following symptoms of head ache, retro orbital pain, myalgia, arthralgia, rash and haemorrhagic manifestation are considered as dengue suspects.

**Dengue confirmed (Seropositive)**

The clinical diagnosis of dengue may be confirmed by laboratory testing by the measurement of an antibody response IgM or IgG and diagnosis by ELISA is considered to be the diagnostic gold standard.

**Health system cost**

Health system cost refers to the cost incurred by the provider. The health system in this study is a public health facility which provides medical services at subsidised rate or free of cost. Thus all medical cost were assumed to be incurred only by the public health facility. This includes Human Resource, Infrastructure, Equipment, Investigation, Treatment and Medication cost.

**Diagnostic test cost**

The test cost incurred per person for dengue diagnosis. In this study screening for dengue was done using Complete blood count (CBC) and confirmation is done for those with <100000 Platelet count by Enzyme Linked Immune Sorbent Assay (ELISA). The per test cost for CBC and ELISA was taken from Central Government Health Scheme (CGHS) investigation rate for public health facility.

**Patient cost**

Non-Medical cost from Hariharan et al study was taken for patient cost.^[[1]](#endnote-1)^ This includes travel cost, food cost, other out of pocket expenditure for non medical goods or services and indirect cost (productivity loss)

**Indirect costs**

Indirect costs refer to the loss of income resulting from the work absenteesim, interruption of normal or preferred activities of patient and household members. The study which is reffered for indirect cost considers 18.8 years average productive life years lost for death patient.^[[2]](#endnote-2)^

**Time Horizon**

When designing a comparative outcomes or a cost-effectiveness analysis, the time horizon defining the duration of time for outcomes assessment considered. The time horizon must be long enough to capture the intended and unintended benefits and harms of the intervention.^[[3]](#endnote-3)^

This study considers life time horizon to capture all cost and effectiveness.

**Decision Tree**

Decision tree is the most powerful and popular tool for classification and prediction. A Decision tree is a flowchart like tree structure, where each internal node denotes a test on an attribute, each branch represents an outcome of the test, and each leaf node (terminal node) holds a class label.

**Cost-effectiveness analysis**

Cost-effectiveness analysis (CEA) is a way to examine both the costs and health outcomes of one or more interventions. It compares an intervention to another intervention (or the status quo) by estimating how much it costs to gain a unit of a health outcome, like a life year gained or a death prevented.

**Incremental cost efectiveness ratio**

The incremental cost effectiveness ratio (ICER) between the standard MDT program with the additional MIP vaccination intervention to newly detected leprosy patient and their contact versus the standard MDT program. The difference in treatment costs with and without the vaccination intervention was calculated and compared with the difference in the number of QALYs gained.

ICER = Cost of the proposed dengue diagnosis strategy (PHC) – Cost of the current dengue

Diagnosis strategy (THC)

____________________________________________________________________

QALY of the proposed dengue diagnosis strategy (PHC) – QALY of the current

Dengue diagnosis strategy (THC)

**Net Monetary Benefit**

Net Monetary benefit (NHB) is a summary statistic that represents the value of an intervention in monetary terms considering a willingness to pay threshold for a unit of benefit. This study considers per capita GDP as willingness to pay threshold

NMB= Cost – (QALY* Willingness to pay Threshold)

Incremental NMB (INMB) measures the difference in NMB between alternative interventions, a positive incremental NMB indicating that the intervention is cost-effective compared with the alternative at the given willingness-to-pay threshold.

INMB= NMB (Proposed Strategy) – NMB (Current Strategy)

**Quality Adjusted Life Year**

The Quality-Adjusted Life Year (QALY) is a standardized measure of disease burden which combines both survival and health-related quality of life into a single index. The QALY is primarily used in cost-effectiveness analyses to guide decisions regarding the distribution of limited health care resources among competing health programs or interventions for a population of interest, but has also been used to aid decisions regarding clinical management and individual patient care.

QALY= Utility * Expected Life years

**One-way sensitivity analysis**

Univariate/one way sensitivity analysis (OSA) is to assess the impact that changes in a certain input (parameter) will have on the output results of an economic evaluation. This will help to assess the robustness of the result to that parameter. It is helpful for decision makers to have insights into the relationship between specific input parameters and the model outputs.

**Probabilistic sensitivity analysis**

Probabilistic sensitivity analysis (PSA) demonstrates the parameter uncertainty in a decision problem. The technique involves sampling parameters from their respective distributions (rather than simply using mean/median parameter values). This technique used in economic modelling that allows the modeller to quantify the level of confidence in the output of the analysis, in relation to uncertainty in the model inputs.

**Cost–effectiveness threshold**

Cost–effectiveness threshold is the ceiling ICER beyond which interventions are no longer considered cost effective, reflecting the maximum value decision makers attach to health benefits. Three general approaches have been used to provide clear guidance to policy makers: (i) thresholds based on per capita national incomes; (ii) benchmark interventions and (iii) league tables. In recent years, the most common approach has involved the use of thresholds based on GDP. Under this approach which has been promoted by the World Health Organization’s Choosing interventions that are cost–effective (WHO-CHOICE) an intervention that costs less than three times the national annual GDP per capita is considered cost–effective, whereas one that costs less than once the national annual GDP per capita is considered highly cost–effective.^^[[4]](#endnote-4)^^ We followed GDP as threshold value.

**Calculations**

1. **CBC test**

| **Parameter** | **Formula** |
| --- | --- |
| Test Positive | Number of cohort population x Probability of True test positive |
| Probability of true positive with CBC (<100000 PLC) | (Prevalence of Dengue x Probability of <100000 PLC) ) + ((1-Prevalence of Dengue) x (1-Probability of <100000 PLC) x Probability of <100000 PLC) |

1. **ELISA test**

| **Parameter** | **Formula** |
| --- | --- |
| Test Positive | Number of cohort population with <100000 platelet count x Probability of ELISA test positive |
| Probability of ELISA test positive | (Prevalence of Dengue x Test sensitivity) + ((1-Prevalence of Dengue) x (1-Test specificity)) |
| True Positive (Positive Predictive value) | (Test sensitivity x Prevalence of Dengue) /  (Test sensitivity x Prevalence of Dengue) + ((1-Test specificity) x (1-Prevalence of Dengue)) |
| True Negative (Negative Predictive value) | ((1-Prevalence of Dengue) x Test specificity) /  (Test specificity x (1-Prevalence of Dengue)) + ((1-Test sensitivity) x Prevalence of Dengue) |

1. **Mortality due to dengue**

| **Parameter** | **Formula** |
| --- | --- |
| Probability of mortality due to DHF | (Probability of mortality due to dengue * 40%) |
| Probability of mortality due to DSS | (Probability of mortality due to dengue * 60%) |
| Relative risk of mortality due to DHF or DSS in proposed strategy | Probability of mortality due to DHF or DSS in proposed strategy/ Probability of mortality due to DHF or DSS in current strategy |

1. **Discount factor calculation**

The discount factor is multiplied with life years (LY), quality adjusted life years (QALY) and cost to obtain discounted LY, QALY and cost.

1 ^ (Expected remaining life years)

**Discount factor = ________________**

**1+Discount rate**

**Discounted Life Years (LY)** = LY* Discount factor

**Discounted Quality adjusted life Years (QALY) =** QALY* Discount factor

**Discounted Cost** = Cost * Discount factor

1. **Cost estimation**

Costs were obtained from meta-analysis report, this did not include diagnostic test cost. Thus, diagnostic test cost was added in cost calculation.

Cost for undifferentiated fever cases who tested negative for dengue (True negative) includes only diagnostic test cost as other costs were unavailable.

1. **Budget Impact Analysis**
2. **Implementation cost**

Cost of haematology analyser, Cost of reagent, Training cost and maintenance cost is considered for implementation cost estimation. Haematology analyser and reagent (that can be used for 3000 samples) cost of three different brands is taken from Government e-marketing (GEM) website and average cost is taken for analysis. Considering installation of one haematology analyser every PHC in Tamil Nadu (n=1421), total capital cost is calculated. Three CBC test per person per year is reflected for reagent cost calculation for the total population covered in the proposed strategy. Every year 10% of the total capital cost is considered for maintenance.

Assuming Rs.2000 for training one staff, total training cost is calculated for providing training to two staff per PHC.

1. **Budget Impact**

Gain in total health benefits (in terms of QALY and severe cases avoided) every year due to proposed strategy when compared with the base year is assessed. It is then plotted in line graph for trend analysis.

Incremental Health Benefit (Year x) = Total health benefit (Year x) – Total health benefit (Base Year)

ICER value is calculated for each year in comparison with base year and ICER plane is drawn for assessing change in the incremental cost-effectiveness over the years.

Minimum population coverage at which the proposed strategy is cost-effective is estimated.

1. D. Hariharan et al. Economic burden of dengue illness in India from 2013 to 2016: A systematic analysis. International Journal of Infectious Diseases 84S (2019) S68–S73. [↑](#endnote-ref-1)
2. Shepard DS, Undurraga EA, Halasa YA, Stanaway JD. The global economic burden of dengue: a systematic analysis. Lancet Infect Dis 2016;16 (August (8)):935–41. [↑](#endnote-ref-2)
3. Basu A, Maciejewski ML. Choosing a Time Horizon in Cost and Cost-effectiveness Analyses. *JAMA.* 2019; 321(11):1096–1097. [↑](#endnote-ref-3)
4. Elliot Marseille, Bruce Larson, Dhruv S Kazi, James G Kahn, Sydney Rosen. Thresholds for the cost–effectiveness of interventions: alternative approaches. Bulletin of the World Health Organization 2015; 93:118-124. [↑](#endnote-ref-4)
